# Modelling HIV/AIDS Epidemic among Men Who Have Sex with Men in China

**DOI:** 10.1155/2013/413260

**Published:** 2013-09-30

**Authors:** Xiaodan Sun, Yanni Xiao, Zhihang Peng, Ning Wang

**Affiliations:** ^1^Department of Applied Mathematics, School of Mathematics and Statistics, Xi'an Jiaotong University, Xi'an 710049, China; ^2^Department of Epidemiology and Biostatistics, Nanjing Medical University, Nanjing 210029, China; ^3^National Center for AIDS/STD Prevention and Control, Chinese Center for Disease Control and Prevention, 155 Changbai Road, Beijing 102206, China

## Abstract

A compartmental model with antiviral therapy was proposed to identify the important factors that influence HIV infection among gay men in China and suggest some effective control strategies. We proved that the disease will be eradicated if the reproduction number is less than one. Based on the number of annual reported HIV/AIDS among MSM we used the Markov-Chain Monte-Carlo (MCMC) simulation to estimate the unknown parameters. We estimated a mean reproduction number of 3.88 (95% CI: 3.69–4.07). The estimation results showed that there were a higher transmission rate and a lower diagnose rate among MSM than those for another high-risk population. We compared the current treatment policy and immediate therapy once people are diagnosed with HIV, and numerical studies indicated that immediate antiviral therapy would lead to few HIV new infections conditional upon relatively low infectiousness; otherwise the current treatment policy would result in low HIV new infection. Further, increasing treatment coverage rate may lead to decline in HIV new infections and be beneficial to disease control, depending on the infectiousness of the infected individuals with antiviral therapy. The finding suggested that treatment efficacy (directly affecting infectiousness), behavior changes, and interventions greatly affect HIV new infection; strengthening intensity will contribute to the disease control.

## 1. Introduction

After the detection of the first acquired immunodeficiency syndrome (AIDS) patient, human immunodeficiency virus (HIV) spreads at an alarming rate worldwide. In recent years, though the number of people newly infected with HIV is decreasing, the prevalence of HIV among men who have sex with men (MSM) has increased significantly in China since 2005. In China, the first AIDS patient among MSM was found in 1989 and the proportion of reported cases resulting from homosexual contact in year 2005 to 2011 is 0.4%, 2.5%, 3.4%, 5.9%, 8.6%, 10.8%, and 13.0%, respectively [1]. The prevalence of HIV among MSM is increasing year by year, has reached an average of 5% in large and medium cities, and is greater than 10% in the main cities of the Southwest [1]. Among all people living with HIV, the proportion of people infected by homosexual contact increases from 14.7% in 2009 to 17.4% in 2011 [2]. In mainland China MSM bear a high burden of HIV since the cultural norm may cause them to wish to be *hidden*, which makes them very likely to be exposed to HIV, and moreover causes much more difficulties to implement intervention measures. At the same time, anal intercourse between men, if unprotected, carries a high risk of HIV transmission [3]. There are studies that showed that HIV infection rate via homosexual contact is much higher than heterosexual contact [4]. So, how to identify the important factors which greatly influence HIV infection among MSM and control the quick increase in HIV epidemic among MSM became urgent in recent years. The primary objective of our study is to understand the HIV epidemic among MSM and to try to suggest some control strategies.

Discovered in 1981, HIV is one of the few things that draw attention from both mathematicians, medical scientists and behavioral scientists. Many models have been proposed in order to predict and control the spread of HIV effectively. A basic SIR model was formulated by Gran et al. [5] in 2008. Considering the feature of chronic disease, the authors generalized the basic model to a Markov model to represent different infection rates in different stages [5]. In such model, HIV/AIDS infected individuals are divided into 13 different stages according to CD4+ level, and at each stage people have a specific infection rate. The idea of dividing the infected group to different stages has been widely used in the study of HIV dynamics [6–10]. In 2008, Zhou et al. [10] proposed a staged discrete model to investigate HIV epidemic in mainland China. We will also utilize the idea in this paper to formulate our mathematical model.

In mainland China, the government initiated a large scale of antiviral therapy since 2003, and free treatment has been expanded to all HIV-positive individuals whose CD4+ T count is less than 350 cells per *μ*L (microliter) of blood from 2007. In recent years, antiviral therapy has become a measure to control the epidemic since there are studies demonstrating that antiviral therapy can extend the life expectancy of HIV/AIDS infected individuals and reduce their infection rates [4, 13, 18, 19]. Granich et al. [12] put forward a new therapy, starting antiviral therapy as soon as people are diagnosed with HIV. And he proved that this therapy can reduce the infection rate and death rate greatly. Note that lower infection rate is beneficial to the disease control, but extended life span increases the risk to infect others. For this high-risk group, whether immediate antiviral therapy once people are diagnosed with HIV is beneficial to disease control or not is controversial since early treatment will inevitably result in early occurrence of drug resistance due to bad adherence and no additional drugs are available [20]. So, to what extent antiviral therapy will be implemented or when to initiate antiviral therapy is unclear. All these fall within the scope of this study.

This paper is divided into 6 sections. In Section 2, a mathematical model with antiviral therapy is formulated following a modeling approach for stratification of the population according to the clinical progression of the disease and epidemiological status of the individuals [6]. In Section 3, the unknown parameters and initial values involved in the model are estimated. In Section 4, the main results of this paper are given which include the threshold dynamics, prediction for the disease, the effects of antiviral therapy, and the effects of intervention measures. In Section 5, some sensitivity analyses are conducted. At last, discussions are given in Section 6.

## 2. Mathematical Model

**Figure 1:**
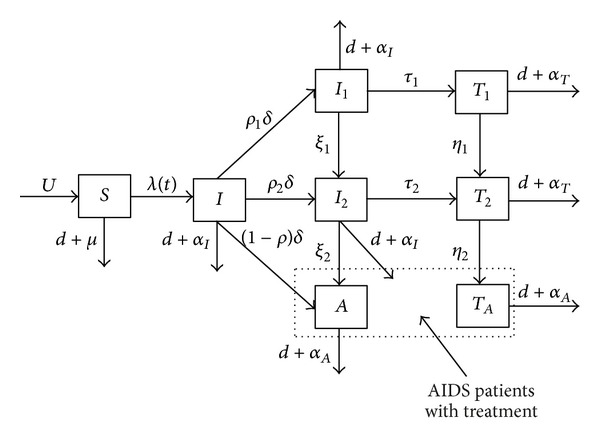
The flow diagram of model with antiviral therapy.

Our model is formulated based on the key epidemiological properties of HIV/AIDS and some implemented public heath interventions such as condom use and antiviral therapy. The underlying structure of the model comprises classes of individuals among MSM who are high-risk susceptibles (*S*), HIV infected but not yet diagnosed (*I*), diagnosed HIV-positive individuals who have not yet progressed to AIDS stage, and those with AIDS clinical symptoms (*A*). Further, according to the CD4+ T cell counts in the blood we divide the diagnosed HIV-positive individuals without receiving treatment into two different stages: the HIV-positive individuals with *l* > 350 (*I*_1_), individuals with 200 < *l* < 350 (*I*_2_), where *l* denotes the CD4+ T level in the blood (i.e., the number of CD4+ T cells per microliter of blood). HIV-positive individuals in group *I*_1_ and *I*_2_ will enter into *T*_1_ and *T*_2_ if they receive antiviral therapy, respectively, and finally progress to the AIDS stage (*T*_*A*_). The flow diagram is described in Figure 1. Let *A* be AIDS patients who are diagnosed after onset of AIDS. Since all AIDS patients are given treatment at the present stage in China, here we do not distinguish between AIDS patients in *A* and those in *T*_*A*_. The high-risk population size is represented by *N*; that is, *N* = *S* + *I* + *I*_1_ + *I*_2_ + *A* + *T*_1_ + *T*_2_ + *T*_*A*_,

(1) $$\begin{array}{c} S^{\prime }=U-\lambda \left(t\right)-\left(d+\mu \right)S,\\ I^{\prime }=\lambda \left(t\right)-\left(d+\alpha_{I}+\delta \right)I,\\ I_{1}^{\prime }=\rho_{1}\delta I-\left(d+\alpha_{I}+\tau_{1}+\xi_{1}\right)I_{1},\\ I_{2}^{\prime }=\rho_{2}\delta I+\xi_{1}I_{1}-\left(d+\alpha_{I}+\tau_{2}+\xi_{2}\right)I_{2},\\ A^{\prime }=\left(1-\rho \right)\delta I+\xi_{2}I_{2}-\left(d+\alpha_{A}\right)A,\\ T_{1}^{\prime }=\tau_{1}I_{1}-\left(d+\alpha_{T}+\eta_{1}\right)T_{1},\\ T_{2}^{\prime }=\tau_{2}I_{2}+\eta_{1}T_{1}-\left(d+\alpha_{T}+\eta_{2}\right)T_{2},\\ T_{A}^{\prime }=\eta_{2}T_{2}-\left(d+\alpha_{A}\right)T_{A}. \end{array}$$

We assume that people enter into the susceptible class at a rate *U*, exit high-risk population at a constant rate *μ*. Each one has a natural death rate (*d*). It is assumed that the transmission probability for the undiagnosed HIV-positive individuals per high-risk behavior (i.e., male-male sex) is a constant, denoted by *β*. *c* represents the contact rate per year, and *π* represents the protection rate by interventions such as condom use and antiviral therapy. Since HIV/AIDS infected individuals in different stages may have different transmission probabilities per high-risk behavior due to different CD4 cell count, antiviral therapy, behavior changes, and so forth, we then introduce a modification factor for each infectious class, denoted by *ε*_*I*_1__, *ε*_*I*_2__, *ε*_*T*_1__, *ε*_*T*_2__, and *ε*_*A*_, respectively. Thus, susceptible people become HIV infected at a rate *S*(*I* + *ε*_*I*_1__ *I*_1_ + *ε*_*I*_2__ *I*_2_ + *ε*_*T*_1__ *T*_1_ + *ε*_*T*_2__ *T*_2_ + *ε*_*A*_(*A* + *T*_*A*_))/*N*. *ρ*_1_ and *ρ*_2_ are the proportions of HIV-positive individuals with *l* > 350 and 200 < *l* < 350 when diagnosed, respectively. Let *ρ* = *ρ*_1_ + *ρ*_2_, and then 1 − *ρ* is the proportion of individuals who have already progressed to AIDS stage when diagnosed. Constants *ξ*_1_ and *ξ*_2_ represent the progression rates from *I*_1_ to *I*_2_ and from *I*_2_ to *A*, respectively. *η*_1_ and *η*_2_ denote the progression rates from *T*_1_ to *T*_2_ and from *T*_2_ to *T*_*A*_, respectively. *α*_*i*_  (*i* = *I*, *T*, *A*) and *τ*_*i*_  (*i* = 1,2) represent the disease related death rate and antiviral therapy coverage rate for each stage. The model equations are given in (1), where *λ*(*t*) = *βc*(1 − *π*)*S*(*I* + *ε*_*I*_1__ *I*_1_ + *ε*_*I*_2__ *I*_2_ + *ε*_*T*_1__ *T*_1_ + *ε*_*T*_2__ *T*_2_ + *ε*_*A*_(*A* + *T*_*A*_))/*N*. The model is illustrated in Figure 1, and definitions of parameters are given in Table 2. We can verify that any solution of system (1) with nonnegative initial value is nonnegative. And it is easy to get that *G* = {(*S*, *I*, *I*_1_, *I*_2_, *A*, *T*_1_, *T*_2_, *T*_*A*_) ∈ *R*_+_^8^ : *S* + *I* + *I*_1_ + *I*_2_ + *A* + *T*_1_ + *T*_2_ + *T*_*A*_ ≤ *U*/*d*} is positively invariant.

## 3. Data and Parameter Estimation

### 3.1. Data

Based on the current surveillance system we can get the number of annual reported HIV-positive cases and AIDS patients among MSM from year 1985 to 2009 in mainland China. The first HIV infected case among MSM in China was reported in 1989. However, the disease has spread very quickly among this population. The proportion of reported cases resulting from homosexual contact increases as follows: 2.5% (2006), 3.4% (2007), 5.9% (2008), and 8.6% (2009) [1]. Sentinel surveillance results have shown that the HIV-positive rates among MSM population in different regions have been consistently greater than 1% and are increasing year by year. Homosexual transmission is becoming one of the most important drivers of the AIDS epidemic. We choose year 2005 as a starting point since the consistent surveillance and testing policy has been implemented in mainland China since then [15].

### 3.2. Parameter Values Obtained from Data or the Literature

The natural death rate was estimated to be *d* = 0.0149 [11]. Generally speaking, if a person is a homoeroticism he tends to be a homoeroticism, through all his life. So we roughly choose *μ* = 1/40  year^−1^. Zhou et al. [10] estimated the progression rate from *I*_1_ to *I*_2_ is *ξ*_1_ = 1/6 and the progression rate from *I*_2_ to *A* is *ξ*_2_ = 1/3. Gran et al. [5] estimated that the scale of progression rate reduced by antiviral therapy is 1/2. So we choose *η*_1_ = 1/2*ξ*_1_ = 1/12 and *η*_2_ = 1/2*ξ*_2_ = 1/6. Because *ξ*_1_ = 1/2∗*ξ*_2_, we can suppose that one-third of the HIV-positive individuals are in stage *I*_2_ and two-thirds are in stage *I*_1_ when diagnosed; that is, *ρ*_1_ = 2*ρ*/3 and *ρ*_2_ = *ρ*/3. Xiao et al. [15] estimated the additional death rate for the diagnosed HIV-positive individuals to be 0.172; then we choose *α*_*I*_ = 0.172. The disease related death rate for AIDS patients without receiving treatment is estimated to be 0.393 by Zhang et al. [17]. The ministry of health, China [16], estimated that the disease related death rate can be decreased by 65.3% after antiviral therapy, so the disease related death rate for AIDS patients with antiviral therapy is *α*_*A*_ = 0.393∗(1 − 65.3%) = 0.136. The disease-related death rate for HIV-positive individuals with antiviral therapy is *α*_*T*_ = 0.172∗(1 − 65.3%) = 0.06.

Recently, antiviral therapy is not provided to HIV-positive individuals with *l* > 350, we then set *τ*_1_ = 0. According to the treatment policy of China, the HIV-positive individuals with *l* > 350; are given treatment since early 2007. Lou et al. [13] and Xu et al. [14] estimated the treatment coverage rate for HIV-positive individuals with 200 < *l* < 350 nowadays to be 0.2. Then we choose time-dependent function *τ*_2_ = 0.2 from year 2007 to 2009 and *τ*_2_ = 0 from year 2005 to 2006. From the database we can get some initial values for the system: *I*_1_(0) = 99, *I*_2_(0) = 49, and *A*(0) = 53. Considering that there are no HIV-positive individuals receiving antiviral therapy in year 2005, thus we have *T*_1_(0) = *T*_2_(0) = *T*_*A*_(0) = 0.

In order to determine the relative infectiousness for each class we follow the principle employed by Gran et al. [5]. They divided the diagnosed HIV-positive individuals into 5 stages based on the CD4+ T cell counts in the blood (above 500, 350–499, 200–349, below 200 copies, and AIDS stage). The numbers of new infections caused by one individual, in each stage, per unit time in a totally susceptible population are 0.031, 0.025, 0.017, 0.013, and 0 for these 5 stages, respectively. Note that we combine the first two stages as one stage in our model. We calculate *ε*_*I*_1__ as follows: the number of new infections in stage *I*_1_ is ((1/0.0455)/(1/0.0455 + 1/0.125)) × 0.031 + ((1/0.125)/(1/0.0455 + 1/0.125)) × 0.025 = 0.0294, where 0.0455 and 0.125 are the progression rates from stage *I*_1_ to stage *I*_2_ and from stage *I*_2_ to stage *I*_3_. By similar method we get that the numbers of new infections in *I* and *I*_2_ are 0.0407 and 0.017, respectively. Then, we have 1 : *ε*_*I*_1__ : *ε*_*I*_2__ = 0.0407 : 0.0294 : 0.017, which implies that *ε*_*I*_1__ = 0.7224, *ε*_*I*_2__ = 0.4177. Other unknown parameters are estimated on the basis of the real data.

### 3.3. MCMC Procedure

We utilize the Markov-Chain Monte-Carlo (MCMC) simulation to estimate the main parameters, and initial values of model (1). Metropolis-Hastings (M-H) algorithm [21–26] are employed for MCMC simulation. The algorithm runs for 210000 iterations with a burn-in of 10000 iterations. Using the rest 200000 samples, we obtain our estimates [27]. The estimation procedure is carried out in two steps. Firstly, in order to reduce the number of parameters that need to be estimated to the minimum, we use the method proposed by Tang et al. [28]. Note that the size of high-risk population is very large compared with HIV/AIDS infected population; *S*/*N* approximately equals 1. Assuming *S* = *N*, we can get a reduced model ((A.1)), given in Appendix A. By fitting the reduced model ((A.1)) to the data on annual reported HIV/AIDS cases from 2005 to 2009, we get the estimates for the transmission coefficient *β*_0_, proportion of diagnosed individuals who are HIV-positive *ρ*, diagnose rate *δ*, modification factors *ε*_*T*_2__, *ε*_*A*_, and initial value of undiagnosed infected individuals *I*(0). Here we assume *ε*_*T*_1__ = *ε*_*T*_2__ since there are no significant difference between the infectiousness of people in these two groups. Using the simulation results of these parameters we can get the Markov Chain of basic reproduction number *R*_0_ and its variation. Secondly, based on the estimation results we repeat the same procedure by fitting model (1) to our real data to estimate the rest of the unknown parameters *S*(0) and *U*. See Appendix A for details about parameter estimation.

## 4. Main Results

### 4.1. Threshold Dynamics

The basic reproduction number *R*_0_, the average number of secondary cases generated by a single primary case in a fully susceptible population during its average infectious period [29, 30], is a threshold parameter for the infectious disease and can help determine whether an infectious disease will spread through a population. Following the next-generation matrix method [29] we can get *R*_0_ for system (1) (see Appendix B for details). For convenience, we let *β*_0_ = *βc*(1 − *π*), *ω*_1_ = *d* + *α*_*I*_ + *δ*, *ω*_2_ = *d* + *α*_*I*_ + *τ*_1_ + *ξ*_1_, *ω*_3_ = *d* + *α*_*I*_ + *τ*_2_ + *ξ*_2_, *ω*_4_ = *d* + *α*_*A*_, *ω*_5_ = *d* + *α*_*T*_ + *η*_1_ and *ω*_6_ = *d* + *α*_*T*_ + *η*_2_. The expression of *R*_0_ is given as follows:

(2) R0=β0ω1[1+ρ1δεI1ω2+ρ1δξ1εI2ω2ω3+ρ2δεI2ω3+ρ1δξ1ξ2εAω2ω3ω4+ρ2δξ2εAω3ω4+(1−ρ)δεAω4+ρ1δτ1εT1ω2ω5+ρ1δτ1η1εT2ω2ω5ω6+ρ1δτ2ξ1εT2ω2ω3ω6+τ2ρ2δεT2ω3ω6+ ρ1δτ1η1η2εAω2ω5ω6ω4+ρ1δξ1τ2η2εAω2ω3ω6ω4+ρ2δτ2η2εAω3ω6ω4].

As the model shows, the primary infected individual will stay at the compartments *I*, *I*_1_, *I*_2_, *A*, *T*_1_, *T*_2_, and *T*_*A*_ (if experiencing the stage) for mean times of 1/*ω*_1_, 1/*ω*_2_, 1/*ω*_3_, 1/*ω*_4_, 1/*ω*_5_, 1/*ω*_6_, and 1/*ω*_4_, respectively. The probabilities of the primary patient to experience each stage are 1, *ρ*_1_ *δ*/*ω*_1_, (*ρ*_1_ *δξ*_1_/*ω*_1_ *ω*_2_) + (*ρ*_2_ *δ*/*ω*_1_), (*ρ*_1_ *δξ*_1_ *ξ*_2_/*ω*_1_ *ω*_2_ *ω*_3_) + (*ρ*_2_ *δξ*_2_/*ω*_1_ *ω*_3_) + ((1 − *ρ*)*δ*/*ω*_1_), *ρ*_1_ *δτ*_1_/*ω*_1_ *ω*_2_, (*ρ*_1_ *δτ*_1_ *η*_1_/*ω*_1_ *ω*_2_ *ω*_5_) + (*ρ*_1_ *δτ*_2_ *ξ*_1_/*ω*_1_ *ω*_2_ *ω*_3_) + (*τ*_2_ *ρ*_2_ *δ*/*ω*_1_ *ω*_3_), and (*ρ*_1_ *δτ*_1_ *η*_1_ *η*_2_/*ω*_1_ *ω*_2_ *ω*_5_ *ω*_6_)+(*ρ*_1_ *δξ*_1_ *τ*_2_ *η*_2_/*ω*_1_ *ω*_2_ *ω*_3_ *ω*_6_) + (*ρ*_2_ *δτ*_2_ *η*_2_/*ω*_1_ *ω*_3_ *ω*_6_). And the transmission coefficients at each stage are *β*_0_, *β*_0_ *ε*_*I*_1__, *β*_0_ *ε*_*I*_2__, *β*_0_ *ε*_*A*_, *β*_0_ *ε*_*T*_1__, *β*_0_ *ε*_*T*_2__, and *β*_0_ *ε*_*T*_*A*__. It is easy to see that *β*_0_/*ω*_1_, *β*_0_ *ε*_*I*_1__/*ω*_2_, *β*_0_ *ε*_*I*_2__/*ω*_3_, *β*_0_ *ε*_*A*_/*ω*_4_, *β*_0_ *ε*_*T*_1__/*ω*_5_, *β*_0_ *ε*_*T*_2__/*ω*_6_, and *β*_0_ *ε*_*T*_*A*__/*ω*_4_ represent the numbers of individuals the primary case infects at each stage, respectively. Using the total probability formula we get the expression of *R*_0_.

Meanwhile, system (1) has a disease-free equilibrium (DFE) *E*_0_ = (*S* ^0^, 0,0, 0,0, 0,0, 0), where *S*_0_ = *U*/(*d* + *μ*). From [29] we can get that when *R*_0_ < 1, the disease-free equilibrium of system (1) is locally stable but unstable when *R*_0_ > 1. We can also prove that *E*_0_ is globally attractive when *R*_0_ < 1 (see details in Appendix C.1). Thus, we get the following theorem.

**Theorem 1**

When *R*_0_ < 1, the disease-free equilibrium of system (1) is globally asymptotically stable, but unstable when *R*_0_ > 1.

Besides the disease-free equilibrium the system has an endemic equilibrium *E*_+_ when *R*_0_ > 1. It is not difficult to get the expression of *E*_+_ = (S*, I*, *I*_1_*, *I*_2_*, A*, *T*_1_*, *T*_2_*, *T*_A_*), where S* = (*U* − *ω*_1_ I*)/(*d* + *μ*), *I*_1_* = *e*_1_ I*, *I*_2_* = *e*_2_ I*, A* = *e*_3_ I*, *T*_1_* = *e*_4_ I*, *T*_2_* = *e*_5_ I*, *T*_A_* = *e*_6_ I*, I* = *U*(*R*_0_ − 1)/(*ω*_1_ *R*_0_ +(*d* + *μ*)(1 + *e*_1_ + *e*_2_ + *e*_3_ + *e*_4_ + *e*_5_ + *e*_6_ − (*ω*_1_/(*d* + *μ*)))), *e*_1_ = *ρ*_1_ *δ*/*ω*_2_, *e*_2_ = (*ρ*_2_ *δ*/*ω*_3_) + (*ρ*_2_ *δξ*_1_/*ω*_2_ *ω*_3_), *e*_3_ = ((1 − *ρ*)*δ*/*ω*_4_) + (*ρ*_2_ *δξ*_2_/*ω*_3_ *ω*_4_) + (*ρ*_1_ *δξ*_1_ *ξ*_2_/*ω*_2_ *ω*_3_ *ω*_4_), *e*_4_ = *ρ*_1_ *δ*(1 + *σ*)*τ*_1_/*ω*_2_ *ω*_5_, *e*_5_ = (*ρ*_2_ *δ*(1 + *σ*)*τ*_2_/*ω*_3_ *ω*_6_) + (*ρ*_1_ *δξ*_1_(1 + *σ*)*τ*_2_/*ω*_2_ *ω*_3_ *ω*_6_) + (*ρ*_1_ *δ*(1 + *σ*)*τ*_1_ *η*_1_/*ω*_2_ *ω*_5_ *ω*_6_), and *e*_6_ = (*ρ*_2_ *δ*(1 + *σ*)*τ*_2_ *η*_2_/*ω*_3_ *ω*_6_ *ω*_4_) +(*ρ*_1_ *δξ*_1_(1 + *σ*)*τ*_2_ *η*_2_/*ω*_2_ *ω*_3_ *ω*_6_ *ω*_4_) + (*ρ*_1_ *δ*(1 + *σ*)*τ*_1_ *η*_1_ *η*_2_/*ω*_2_ *ω*_5_ *ω*_6_ *ω*_4_).

Moreover, according to the persistence theorem developed by Smith and Zhao in [31] we can prove the system is uniformly persistent if *R*_0_ > 1. To this end we verify that invariant sets in the boundary of the feasible region are not attractors (see details in Appendix C.2.) Hence we have the following theorem.

**Theorem 2**

System (1) is uniformly persistent when *R*_0_ > 1, in the sense that there is a positive number *δ*_0_ such that for all initial values (*S*_0_, *I*_0_, *I*_1_0__, *I*_2_0__, *A*_0_, *T*_1_0__, *T*_2_0__, *T*_*A*_0__) ∈ *R*_+_ × Int(*R*_+_^7^), the solution of system (1) satisfies

(3) liminf⁡t→∞(S(t),I(t),I1(t),I2(t),A(t),T1(t),T2(t),TA(t))  ≥(δ0,δ0,δ0,δ0,δ0,δ0,δ0,δ0).

### 4.2. Prediction for the Disease

**Figure 2:**
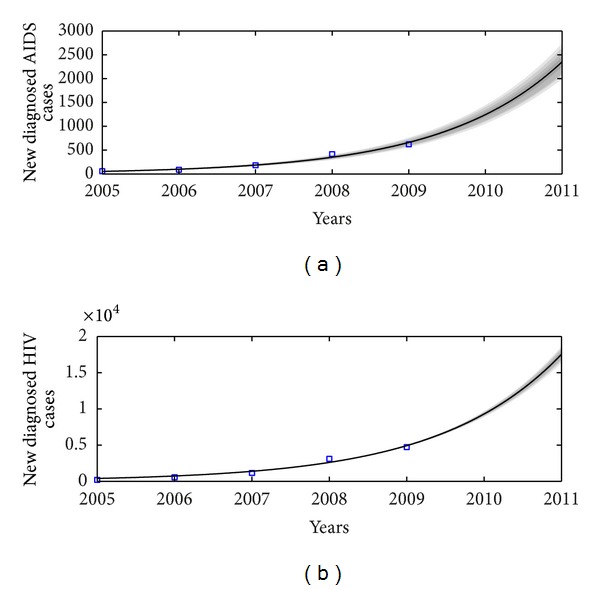
Plots of data fitted results. (a) The number of annual reported AIDS patients. (b) The number of annual reported HIV-positive individuals. Squares denote the real data. Areas from light to dark mean the 50%, 90%, 95%, and 99% predictive probability limits due to parameter uncertainties.

**Figure 3:**
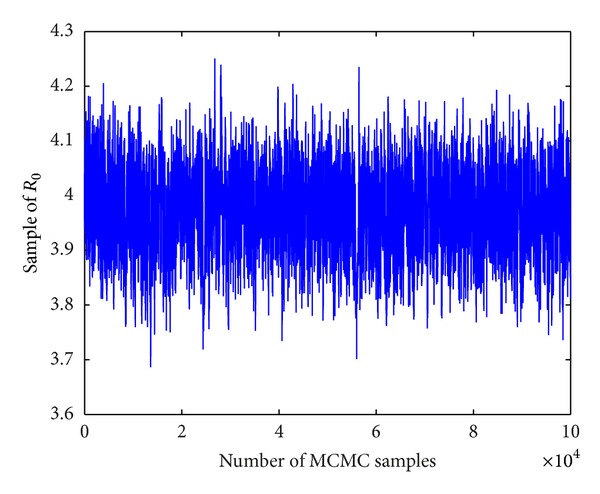
MCMC plots for *R*_0_.

Based on the number of individuals living with HIV (not AIDS) or AIDS among MSM from year 2005 to year 2009, we estimate mean values of parameters and their standard deviations which are listed in Table 2. We also derive the goodness of fit with data together with the uncertainties (shown in Figure 2). The areas from light to dark mean the 50%, 90%, 95%, and 99% limits of the posterior uncertainty due to model parameters. In particular, we get the mean value of the basic reproduction number to be 3.8840 with a estimation error 0.097, and the 95% confidence interval is [3.69,4.07].  Figure 3 describes the Markov Chain for *R*_0_, which has a good convergency. From the estimation results we find that the value of transmission coefficient *β*_0_ is larger than the estimation for heterosexual transmission by Xu et al. [14] and that for the general high-risk population without considering transmission routs by Xiao et al. [15]. This implies that, on the one hand, HIV transmission probability among MSM are higher than that for other high-risk groups [4]; on the other hand, the condom use rate or other interventions among MSM are a bit lower [32]. What is more, the diagnose rate among MSM estimated is only 0.08, which is lower than that for other high-risk groups. That is almost due to the physiological specificity of MSM and the cultural norm of China. A study for Beijing was given in 2006, which pointed out that testing and awareness of HIV infection among MSM are very lacking [33].

**Figure 4:**
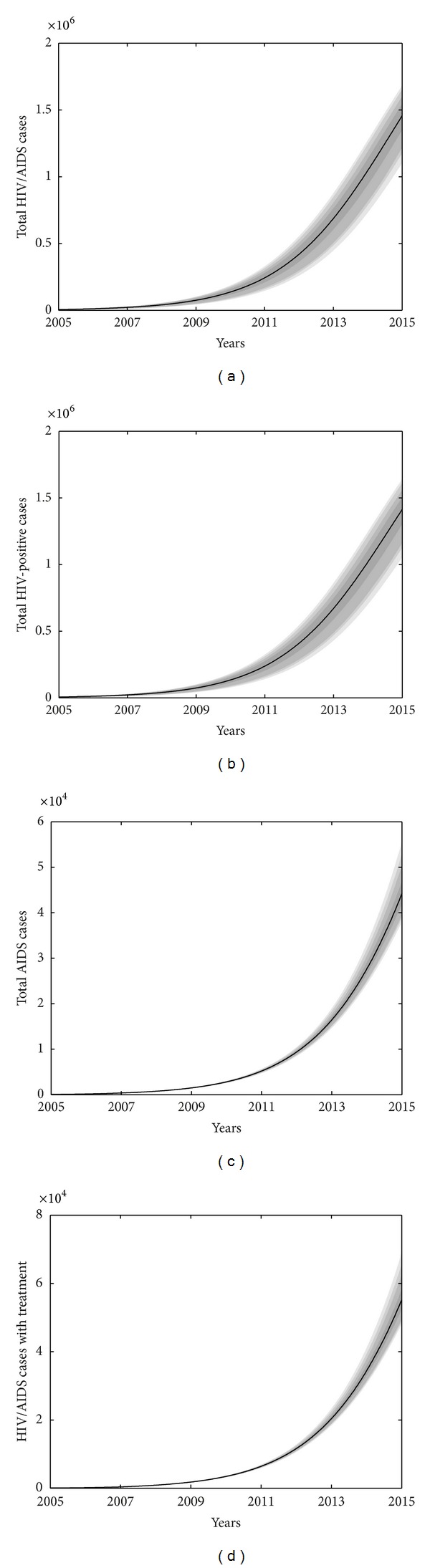
Prediction of HIV/AIDS among MSM in China from 2005 to 2020 and the uncertainties of the model. Areas from light to dark mean the 50%, 90%, 95%, and 99% predictive probability limits due to parameter uncertainties. (a) Total HIV/AIDS cases. (b) Total HIV-positive cases. (c) Total AIDS cases. (d) Total HIV/AIDS cases with antiviral therapy. Parameters and initial values used are shown in Table 2. Antiviral therapy started when CD4+ counts are less than 350 cells per *μ*L.

We suppose that the treatment coverage rate will not change and the current treatment policy will remain the same (i.e., antiviral therapy starts when CD4+ T cell counts are less than 350 per *μ*L of blood). Our estimation shows that the total number of HIV/AIDS individuals among MSM in 2015 will reach 1.46 × 10^6^, with 1.41 × 10^6^ HIV-positive individuals and 4.41 × 10^4^ AIDS patients if the current surveillance, testing, and interventions are unchanged (shown in Figure 4(a)). In particular, our estimation gives that there are 6596 HIV/AIDS infected individuals among MSM receiving antiviral therapy in 2011, which takes up to 55% of the diagnosed individuals among MSM with CD4+ T count less than 350 cells per *μ*L, whereas, estimation from Ministry of Health, people's republic of China stated that the antiviral therapy coverage rate has reached 73.5% in 2011 [2]. This difference agrees with the conclusion obtained by Tong that the treatment coverage rate for HIV/AIDS cases infected sexually is lower than that for HIV/AIDS individuals infected by other routes [34].

### 4.3. Effects of Antiviral Therapy

Antiviral therapy can effectively decrease the viral load in the blood of HIV/AIDS infected individuals, thereby reduceing their infectivity. However, the decrease in viral load consequently alleviates the symptom of HIV/AIDS infected individuals; then they may become active or increase their high-risk behaviors. There are some studies showed that HIV infected individuals may increase their high-risk behaviors since they believe that antiviral therapy can make them more healthy [4, 35–37]. Moreover, antiviral therapy can prolong the lifespan of HIV/AIDS infected individuals, which consequently makes them have more opportunities to infect others. Some researchers supported the conclusion that immediate antiviral therapy once people are diagnosed with HIV is more effective for reducing the infection rate and death rate, and finally controlling the infection [12], whereas other researchers worried that early initiating treatment inevitably induces early occurrence of drug resistance due to poor drug adherence and side effect and consequently results in the decline in treatment efficacy hence it is harmful to control HIV infection [20]. So when antiviral therapy should be started is still controversial and needs a further study. In order to investigate this issue we consider the following two situations and compare the basic reproduction numbers of these two situations.

*Situation 1*. Start antiviral therapy when CD4+ T counts are less than 350 cells per *μ*L of blood (keep the current treatment policy). Then we have *τ*_1_ = *η*_1_ = 0. Let *ω*_2_* = *d* + *α*_*I*_ + *ξ*_1_; we get the expression of basic reproduction number in this situation, which is denoted by *R*_0_*; then

(4) R0∗=β0ω1[1+ρ1δεI1ω2∗+ρ1δξ1εI2ω2∗ω3+ρ2δεI2ω3+ρ1δξ1ξ2εAω2∗ω3ω4+ρ2δξ2εAω3ω4+(1−ρ)δεAω4+ρ1δτ2ξ1εT2ω2∗ω3ω6+τ2ρ2δεT2ω3ω6+ρ1δξ1τ2η2εAω2∗ω3ω6ω4+ρ2δτ2η2εAω3ω6ω4].

*Situation 2*. Start antiviral therapy immediately once people are diagnosed with HIV. In this situation, the expression of basic reproduction number *R*_0_ is given in Section 4.1.

Using the parameter values listed in Table 2 we get that *R*_0_ = 3.93, *R*_0_* = 3.88. This implies that immediate antiviral therapy once diagnosed may cause more new infections and is not beneficial to the disease control if parameter values are chosen as those listed in Table 2. In such scenarios, the effect of expanded lifespan may overweight the effect of reduced infectiousness caused by early treatment.

It is interesting to note that the value of *R*_0_ is greater than *R*_0_* based on the parameter values shown in Table 2. However, immediate antiviral therapy may not always lead to a greater value of the reproduction number. In fact, the relationship between *R*_0_ and *R*_0_* depends on many factors such as treatment coverage, infectiousness, and so forth. In the following we investigate the relation between *R*_0_ and *R*_0_*:

(5) R0−R0∗ =β0ρ1δω1[(εI1+ξ1εI2ω3+ξ1ξ2εAω3ω4+τ2ξ1εT2ω3ω6+ξ1τ2η2εAω3ω6ω4)×(1ω2−1ω2∗)+τ1εT1ω2ω5+τ1η1εT2ω2ω5ω6+ τ1η1η2εAω2ω5ω6ω4].

In order to determine the sign of *R*_0_ − *R*_0_*, we turn to consider

(6) ω1ω2(R0−R0∗)β0ρ1δτ1 =−1ω2∗(εI1+ξ1εI2ω3+ξ1ξ2εAω3ω4+τ2ξ1εT2ω3ω6+ξ1τ2η2εAω3ω6ω4)  +εT1ω5+η1εT2ω5ω6+η1η2εAω5ω6ω4 =−1ω2∗(εI1+ξ1εI2ω3)+1ω5εT1  +(η1ω5−τ2ξ1ω2∗ω3)(εT2+η2ω4ω6εA).

It is obvious that sign⁡(*R*_0_ − *R*_0_*) = sign⁡(*ω*_1_ *ω*_2_(*R*_0_ − *R*_0_*)/*β*_0_ *ρ*_1_ *δτ*_1_), where sign⁡(*a*) denotes the sign function and indicates the sign of *a*. So if *ε*_*T*_1__ and *η*_1_ satisfy the following:

(7) $$\begin{array}{c} \frac{\varepsilon_{T_{1}}}{\omega_{5}}+\left(\frac{\eta_{1}}{\omega_{5}}-\frac{\tau_{2}\xi_{1}}{\omega_{2}^{*}\omega_{3}}\right)\left(\varepsilon_{T_{2}}+\frac{\eta_{2}\varepsilon_{A}}{\omega_{4}\omega_{6}}\right)<\frac{1}{\omega_{2}^{*}}\left(\varepsilon_{I_{1}}+\frac{\xi_{1}\varepsilon_{I_{2}}}{\omega_{3}}\right), \end{array}$$

we have *R*_0_ − *R*_0_* < 0, which means that immediate antiviral therapy once people are diagnosed with HIV will result in a lower value of basic reproduction number and hence lead to fewer new infections.

**Figure 5:**
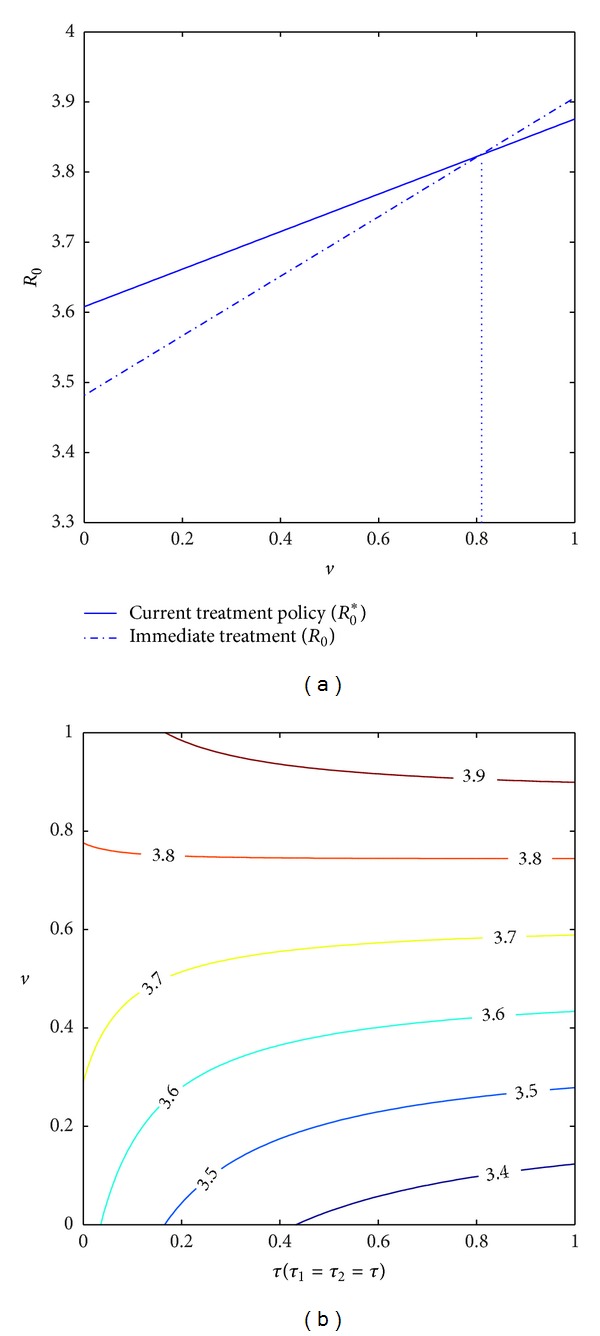
(a) Plots of *R*_0_* and *R*_0_ against factor *v*. (b) Contour plot of *R*_0_ varies the factor *v* and treatment coverage rate, where *τ*_1_ = *τ*_2_ = *τ*. Parameters used are shown in Table 2.

From (7) we obtain that the difference between *R*_0_ and *R*_0_* is determined by many parameter values. For simplicity, we introduce a factor *v* which varies from 0 to 1 to let the modification factors vary simultaneously. To this end, let *v* multiply *ε*_*T*_1__, *ε*_*T*_2__, and *ε*_*A*_; we then analyze the value of *R*_0_* and *R*_0_ as a function of *v*. Thus, *v* = 0 denotes that the infected individuals are not infectious anymore and *v* = 1 means the modification factors *ε*_*T*_1__, *ε*_*T*_2__, and *ε*_*A*_ take values as those listed in Table 2. In order to compare the two situations (antiviral therapy started when CD4+ count less than 350 cells per *μ*L, antiviral therapy started immediately once people are diagnosed with HIV) we draw *R*_0_* and *R*_0_ in Figure 5(a). It shows that there is a critical value v* = 0.81 such that *R*_0_ < *R*_0_* for *v* < v* and *R*_0_ > *R*_0_* for *v* > v*. That is to say, if we can reduce the modification factors to 81%, immediate antiviral therapy will be more beneficial in terms of inducing a less reproduction number. Summing up the above, if the modification factors for people with antiviral therapy are relatively small, the earlier to start antiviral therapy the better; in contrast, if the modification factors are relatively large, postponing antiviral therapy is better in terms of causing relatively few new infections.

*Should We Increase the Treatment Coverage Rate? *In the following we investigate whether increasing the treatment coverage rate is beneficial or harmful to control HIV epidemic. To this end, we examine the variation in *R*_0_ with treatment coverage rate. We simply suppose *τ*_1_ = *τ*_2_ = *τ*. Solving the partial derivative of *R*_0_ with respect to *τ* we have

(8) ∂R0∂τ=β0ω1[−ρ1δω22εI1−(ρ2δω32+ρ1δξ1(ω2+ω3)ω22ω32)εI2+ AεT1+BεT2+CεA],

where

(9) $$\begin{array}{c} A=\frac{\rho_{1}\delta \left(d+\alpha_{I}+\xi_{1}\right)}{\omega_{2}^{2}\omega_{5}}, \end{array}$$

(10) B=ρ1δη1(d+αI+ξ1)ω22ω5ω6+ρ2δ(d+αI+ξ2)ω32ω6+ρ1δξ1ω2ω3ω6−ρ1δξ1τ(ω2+ω3)ω22ω32ω6,

(11) C=−ρ2δξ2ω32ω4−ρ1δξ1ξ2(ω2+ω3)ω22ω32ω4+ρ1δη1η2(d+αI+ξ1)ω22ω4ω5ω6+ρ2δη2(d+αI+ξ2)ω32ω4ω6+ρ1δξ1η2ω2ω3ω4ω6−ρ1δξ1η2τ(ω2+ω3)ω22ω32ω4ω6.

We can verify that *A* > 0,  *B* > 0 (see Appendix D for details about the proof of *B* > 0). Let ∂*R*_0_/∂*τ* = 0; we have

(12) AεT1+BεT2  =ρ1δω22+[ρ2δ+ρ1δξ1(ω2+ω3)ω22]εI2ω22−CεA≜E−.

Thus, there exists a critical value $\overset{-}{E}$ for a combination of parameters *η*_1_, *η*_2_, *α*_*T*_, and so forth such that ∂*R*_0_*/∂*τ*_2_ < 0 for $A\varepsilon_{T_{1}}+B\varepsilon_{T_{2}}<\bar{E}$ and ∂*R*_0_*/∂*τ*_2_ > 0 for $A\varepsilon_{T_{1}}+B\varepsilon_{T_{2}}>\bar{E}$. Note that *A* and *B* are both positive, which implies that if the modification factors for HIV-positive individuals are relatively small, then a large treatment coverage rate will lead to decline in new infections and hence is beneficial to disease control; otherwise increasing treatment coverage rate would result in an increase in new infections. That is to say, given unchanged behavior of HIV-positive individuals, the infectiousness due to antiviral therapy plays a vital role in determining HIV new infections. In particular, when infectiousness due to antiviral therapy is below a critical value, increasing treatment coverage rate is effective to reduce the new infections. Whereas infectiousness is greater than a critical value, increasing treatment coverage rate induces more new infections, which implies the more therapy for the HIV infected individuals, the more new infections produced (see Figure 5(b) for details). It follows from Figure 5(b) that *R*_0_ decreases as treatment uptake rate *τ* increases if *v* is less than a critical value (around 0.6), which implies that increasing treatment coverage is beneficial to disease control in the case of better treatment efficacy; otherwise, increasing treatment is harmful to it. 

### 4.4. Effects of Intervention Measures

In recent years many intervention measures have been implemented to control the quick increase of HIV epidemic among MSM. The intervention measures include (1) strengthening education to the high-risk population, which decreases the constant recruitment rate *U*, the contact rate per year *c* and increases the condom use rate; (2) increasing surveillance and testing, which results in an increase in the diagnose rate *δ*. Note that both decreasing in contact rate *c* and increasing in condom use rate *π* can lead to the decline of transmission coefficient *β*_0_. 

**Figure 6:**
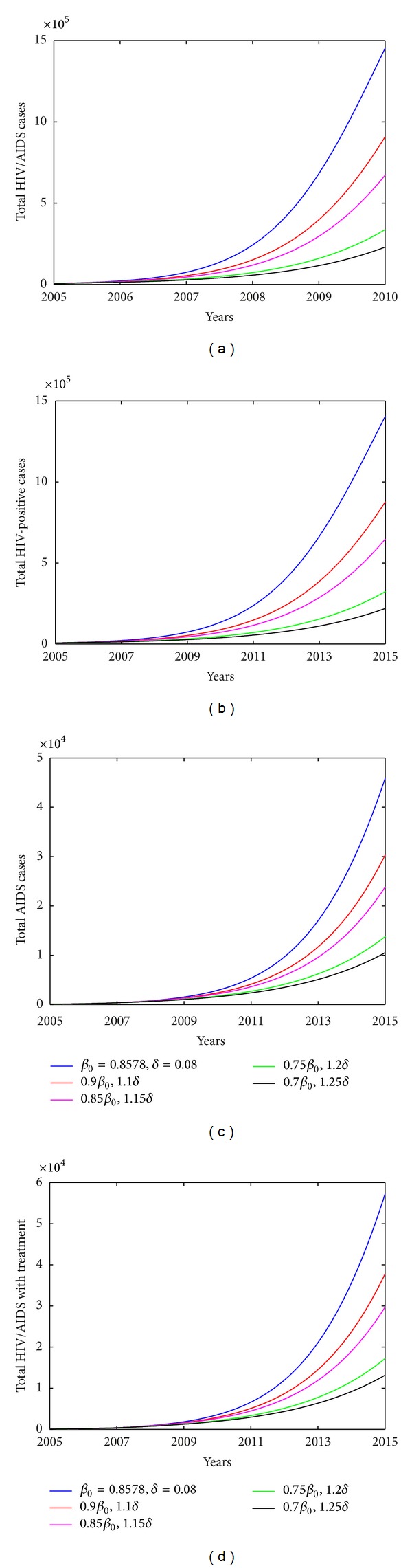
Plots of estimated number of HIV/AIDS cases vary with transmission coefficient *β*_0_ and diagnose rate *δ*. (a) Total number of HIV/AIDS cases. (b) Total HIV-positive cases. (c) Total AIDS cases. (d) Total HIV/AIDS with treatment. Other parameters used are shown in Table 2.

**Figure 7:**
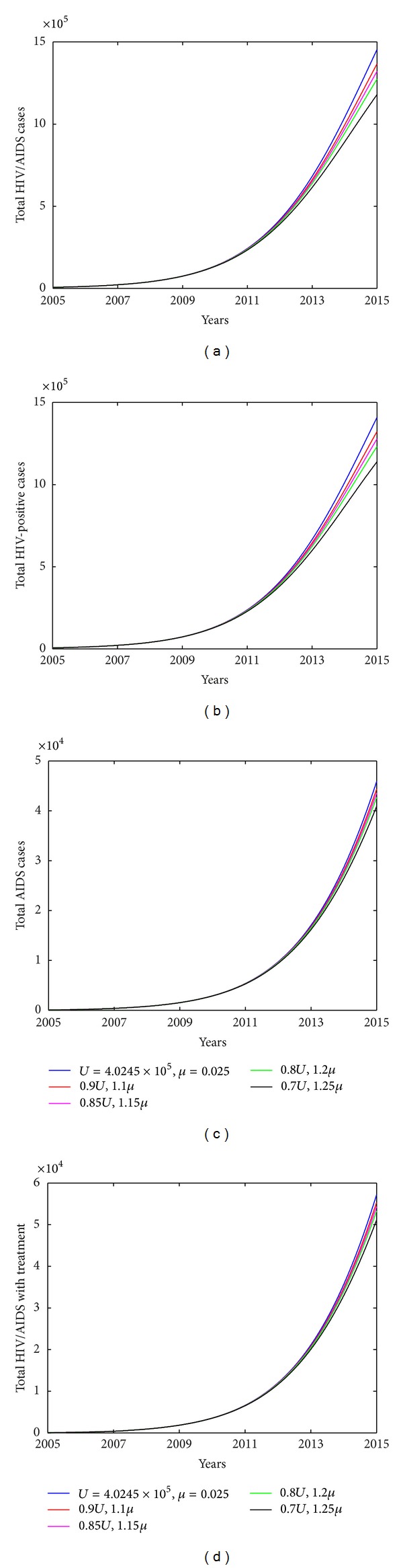
Plots of estimated number of HIV/AIDS cases vary with constant recruitment *U* and exit rate *μ*. (a) Total number of HIV/AIDS cases. (b) Total HIV-positive cases. (c) Total AIDS cases. (d) Total HIV/AIDS with treatment. Other parameters used are shown in Table 2.

**Figure 8:**
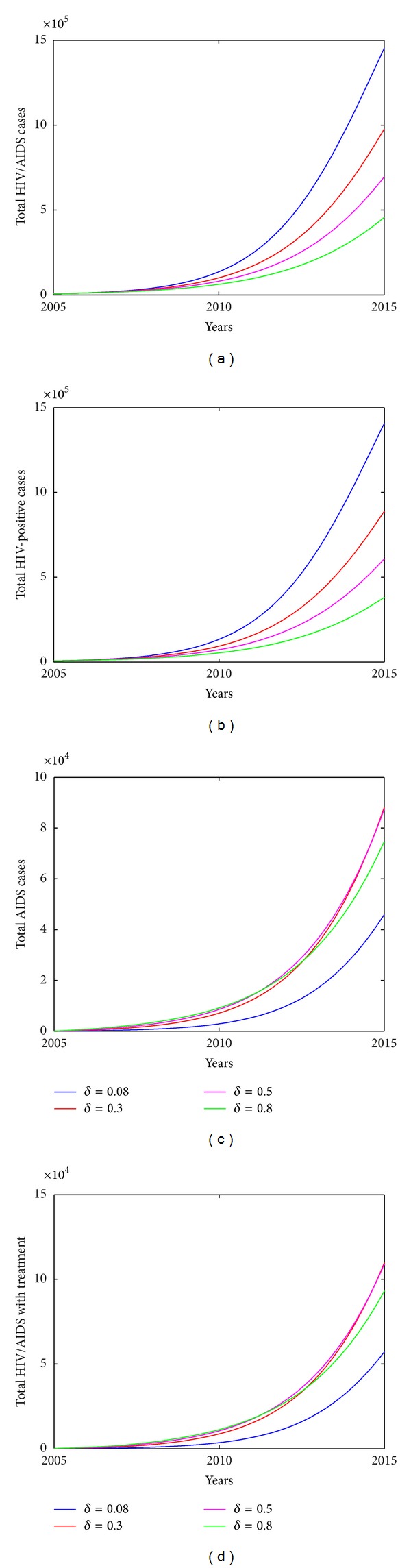
Plots of estimated number of HIV/AIDS cases vary with diagnose rate *δ*. (a) Total number of HIV/AIDS cases. (b) Total HIV-positive cases. (c) Total AIDS cases. (d) Total HIV/AIDS with treatment. Other parameters used are shown in Table 2.

To address the impact of each intervention measure on HIV infection among MSM, we investigate variation in number of HIV/AIDS infected individuals with varying transmission coefficients, treatment uptake rate.  Figure 6 shows that strengthening education to high-risk population (i.e., smaller *β*_0_) and increasing surveillance and testing (i.e., larger *δ*) slow down the spread of HIV significantly. In particular, if *β*_0_ decreases by 30% and *δ* increases by 25%, the total number of HIV/AIDS infected individuals among MSM will decrease to 2.2959 × 10^5^, which is decreased by 84.2%. Similarly, we could increase the diagnose rate *δ* only to examine the effect of improving HIV diagnose and testing strategy on HIV transmission among MSMs. In particular, if the diagnose rate *δ* increases to 0.3, 0.5, and 0.8, the total number of HIV/AIDS infected individuals will decrease by 32.74%, 52.14%, and 68.59% in year 2015, respectively, and the number of individuals with treatment will increase by 92.07%, 90.06%, and 63.00% in year 2015, as shown in Figure 8. It is interesting to note that relatively large testing rate leads to a slow increase of the number of individuals with treatment (shown in Figure 8(d)). Meanwhile, low recruitment rate *U* and large exit rate *μ* will also slow down the spread of HIV however their effects are very limited (as shown in Figure 7). This is because the number of HIV infected individuals is very small compared with the total high-risk population among MSM; thus the variation of *S*/*N* is very limited. This conclusion is in agreement with that for general high-risk population obtained by Xiao et al. [15].

**Figure 9:**
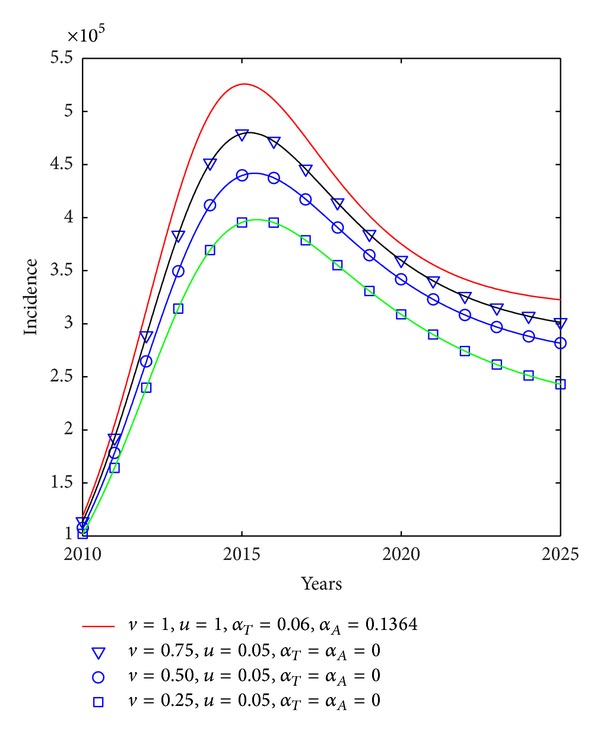
Plots of incidence against factor *v* when antiviral therapy started immediately after diagnose. *τ*_1_ = *τ*_2_ = 0.8. Other parameters used are shown in Table 2.

In order to investigate the effect of antiviral therapy, we study the variation in the incidence of HIV with different antiviral therapy efficacy represented by infectiousness (*ϵ*_*T*_1__, *ϵ*_*T*_2__, *ϵ*_*A*_), disease progression rates (*η*_1_, *η*_2_), and disease-related death rates (*α*_*T*_ and *α*_*A*_). In order to reduce the number of variables we introduce two factors *v*  (0 < *v* ≤ 1) and *u*  (0 < *u* ≤ 1) to describe variation in infectiousness and disease progression rates, respectively. During the analysis, *v* is set to be 0.75, 0.5 and 0.25 to represent that infectiousness is decreased to 75%, 50%, and 25%, respectively. We suppose that antiviral therapy decreased the progression rates to 5%.  Figure 9 shows that HIV incidence will peak in around year 2016, and the better drug efficacy the lower HIV incidence. It indicates that although the drugs are persistently effective, HIV incidence will increase in the recent years. That is because effect of antiviral therapy on HIV epidemic between hosts is not instantaneous but delays some time.

## 5. Sensitivity Analysis

Note that in our model some parameters are known with uncertainties or have large variances, which may greatly affect outcomes. It is then necessary to do the uncertainty and sensitivity analysis such that the sensitive parameters can be detected. To examine the sensitivity of our results to parameter variations, we use latin hypercube sampling (LHS) and partial rank correlation coefficients (PRCCs) [38] to examine the dependence of the reproduction number *R*_0_ and the expected number of total HIV/AIDS individuals in 2015 on each parameter.

**Figure 10:**
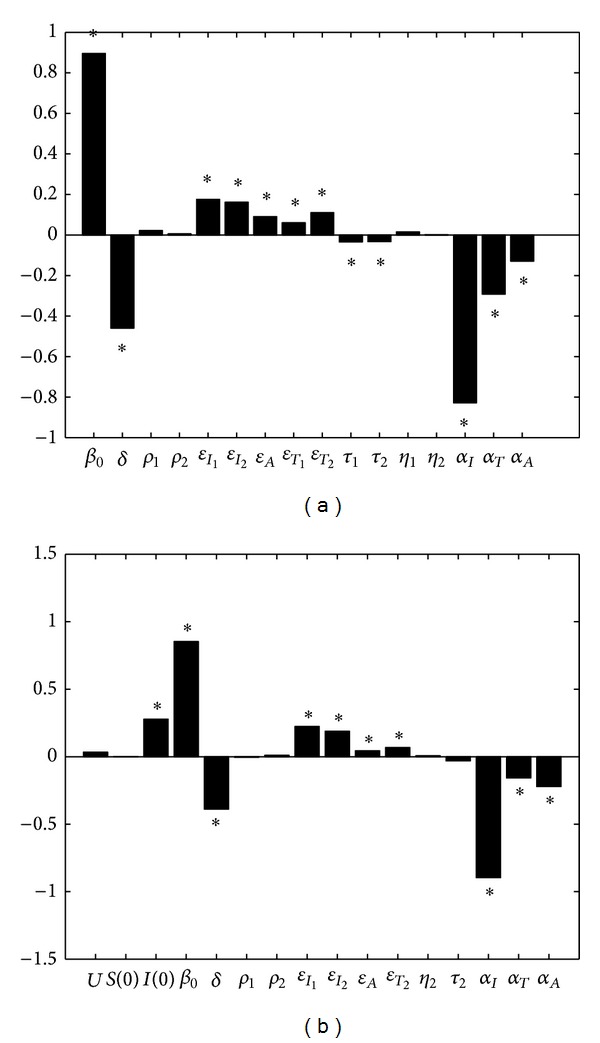
(a) Partial rank correlation coefficients (PRCC) results for the dependence of *R*_0_ on each parameter. (b) Partial rank correlation coefficients (PRCC) results for the dependence of total HIV/AIDS cases in year 2015 on each parameter. ∗ denotes the value of PRCC is not zero significantly, where the significance level is 0.05.

We initially examine the sensitivity of basic reproduction number *R*_0_ to parameter variations. We choose the sample size *n* = 4000, parameters interested as the input variables, and the value of *R*_0_ as the output variable. Parameter values and ranges are listed in Table 1.  Figure 10(a) shows the PRCC value of each parameter. Parameters with star above the bar are the significant ones, and the significance level we choose here is *α* = 0.05. It shows that *β*_0_, *ε*_*I*_1__, *ε*_*I*_2__, *ε*_*A*_, *ε*_*T*_1__, and *ε*_*T*_2__ have positive impact upon *R*_0_, whilst *δ*, *τ*_1_, *τ*_2_, *α*_*I*_, *α*_*T*_, and *α*_*A*_ have negative impact. From the PRCC results we know that *R*_0_ is not sensitive to parameters *ρ*_1_, *ρ*_2_, *η*_1_, and *η*_2_. So, assumptions of *ρ*_1_ = 2*ρ*/3, *ρ*_2_ = *ρ*/3, and *η*_1_, *η*_2_ being half of *ξ*_1_ and *ξ*_2_ are justified and hence have little influence on our main results. Further, we can get that the most influential parameters are *β*_0_, *α*_*I*_ since the PRCC values of them are larger than 0.8. In particular, simple calculation indicates that the basic reproduction number can decrease to less than 1 if *β*_0_ reduces to 0.2.

We also investigate the sensitivity of the expected number of people living with HIV/AIDS to parameter variations. Given the current treatment policy, parameters described in Table 1 except for *ϵ*_*T*_1__, *τ*_1_, and *η*_1_ are chosen as the input variables. The total HIV/AIDS cases in year 2015 is output variable. The PRCC values are shown in Figure 10(b), from which we can find that both *U* and *S*(0) have little impacts on the number of people living with HIV/AIDS in year 2015. The influences of other parameters on total HIV/AIDS cases are similar to their influences on *R*_0_.

## 6. Discussion

According to the CD4+ T cell counts in the blood, we divided the HIV-positive individuals to several stages and formulated a mathematical model with antiviral therapy. The unknown parameters involved in this model were estimated using the MCMC simulation basing on the real data (i.e., the number of annual reported HIV/AIDS among MSM). We defined the threshold value (the basic reproduction number *R*_0_) which determines whether the epidemic goes to extinction or not. By using the stability theories and methods of ordinary differential equation, we proved that the disease-free equilibrium is globally asymptotically stable when *R*_0_ < 1, whilst the system is uniformly persistence when *R*_0_ > 1.

We studied the effect of antiviral therapy in two situations: antiviral therapy started immediately once people are diagnosed with HIV and antiviral therapy started when CD4+ T counts are less than 350 cells per *μ*L (current policy). We found that, given unchanged behaviors after treatment, there exists a critical value for the infectiousness below which immediate treatment is better than the current policy in terms of the reproduction number, whereas current policy exhibits better results than immediate treatment if the infectiousness is greater than the critical level. It indicates that if the treatment efficacy is really good (i.e., relatively low infectiousness), our conclusion suggests immediate treatment; otherwise the current policy is recommended. Similarly, when the infectiousness is relatively low (relatively good treatment efficacy), increasing treatment coverage will decrease the reproduction number and lead to decline in new HIV infection, whilst increasing treatment coverage will result in an increase in new HIV infection for the relatively great infectiousness, which is in agreement with that for heterosexual transmission [14]. Summing up the above, if treatment efficacy is relatively good, our conclusions suggest immediate treatment with high uptake rate; otherwise the current policy is reasonable.

Using the data on the number of new reported HIV/AIDS infected individuals by year among MSM, we obtained estimates of the reproduction number, intervention parameter values, and the high-risk population size. Our estimated reproduction number is 3.88 (95% CI 3.69–4.07) which is in the ranges of estimates for Western Germany (3.43–4.08) and UK (3.38–3.96) [39]. From the estimated parameters we know that the transmission coefficient *β*_0_ is much larger than the estimation for heterosexual transmission [14] and general high-risk population [28]. This result is associated with the conclusion by Lou et al. [13] that MSM are 19 times more likely to be infected with HIV than general population. In fact, there are a lot of money boys in China, also called male sex workers especially in bathhouse, bars, and clubs [34, 40]. Recent surveys showed that the condom use rate among MSM is very low, only less than 30 percent [32]. Anotherstudy showed that anal intercourse between men, if unprotected, carries a high-risk of HIV transmission [3]. All these factors lead to the high probability for HIV transmission among MSM, which is in agreement with our estimation. Our estimation also shows that the diagnose rate among MSM is much lower than that for other high-risk population [15]. Meanwhile, we estimated that the antiviral therapy coverage rate among MSM in 2011 is less than the estimation by Ministry of Health, People's Republic of China [2]. This agrees with the conclusions obtained by Tong [34] that the antiviral therapy coverage rate for individuals infected sexually is lower than other infected groups.

Simulation results show that strengthening education to high-risk population and increasing surveillance and testing can slow down the spread of disease. Further, sensitivity analysis implies that the most influential parameters are infection rate *β*_0_ and disease related death rate for HIV-positive individuals *α*_*I*_. Note that high efficacy drug can reduce the transmission probability of HIV per high-risk behavior [18], and the education may reduce the contact rate and increase the condom use rate. This means that a high effective drug and timely education may effectively control HIV epidemic.

In this paper, we concluded that if the infectiousness for HIV/AIDS infected cases is relatively small, treatment started immediately once diagnosed is more beneficial to disease control. It should be mentioned that we have not considered costs of antiviral therapy. However, early antiviral therapy will increase the financial burden of the government of China and may increase high risk of occurrence of drug resistance. We will consider these factors in the future study.

## Figures and Tables

**Table 1:** Parameter values and ranges.

Parameters	Ranges	Initial values	Parameters	Ranges	Initial values
*U*	[10000, 100000]	402450	*S*(0)	[70000, 150000]	763240
*β*_0_	[0.1, 1]	0.8578	*δ*	[0.01, 1]	0.0816
*ρ*_1_	[0.01, 1]	0.5879	*ρ*_2_	[0.01, 1]	0.2939
*η*_1_	[0.02, 1]	1/12	*η*_2_	[0.02, 1]	1/6
*τ*_1_	[0.02, 1]	0.2	*τ*_2_	[0.02, 1]	0.2
*ϵ*_*I*_1__	[0.01, 1]	0.7224	*ϵ*_*I*_2__	[0.01, 1]	0.4177
*ϵ*_*A*_	[0.01, 1]	0.2495	*α*_*I*_	[0.02, 1]	0.172
*ϵ*_*T*_1__	[0.01, 1]	0.4177	*α*_*T*_	[0.02, 1]	0.06
*ϵ*_*T*_2__	[0.01, 1]	0.3507	*α*_*A*_	[0.02, 1]	0.1364

**Table 2:** Parameters and initial values.

Parameters	Definition	Value	Std	Source
*β*	Transmission probability of HIV per high-risk behavior	—	—	—
*c*	Contact rate per year	—	—	—
*π*	Protection rate by intervention measures (condom use)	—	—	—
*β*_0_	Transmission coefficient, *β*_0_ = *βc*(1 − *π*)	0.8578	0.013	MCMC
*ϵ*_*I*_1__	Modification factor for HIV infected individuals with *l* > 350	0.7224	—	[5]
*ϵ*_*I*_2__	Modification factor for HIV infected individuals with 200 < *l* < 350	0.4177	—	[5]
*ϵ*_*T*_1__	Modification factor for HIV infected individuals with *l* > 350 and receiving antiviral therapy	0.3507	—	—
*ϵ*_*T*_2__	Modification factor for HIV infected individuals with 200 < *l* < 350 and receiving antiviral therapy	0.3507	0.028	MCMC
*ϵ*_*A*_	Modification factor for AIDS patients with antiviral therapy	0.2495	0.028	MCMC
*U*	Recruitment rate of susceptible	402450	20764	MCMC
*d*	Natural death rate	0.0149	—	[11]
*μ*	Exit rate of susceptible	0.025	—	—
*δ*	Diagnose rate	0.0799	0.020	MCMC
*ρ*	Proportion of diagnosed HIV-positive individuals *ρ* = *ρ*_1_ + *ρ*_2_	0.8820	0.006	MCMC
*ρ*_1_	Proportion of diagnosed HIV-positive individuals with *l* > 350	0.5879	—	[10]
*ρ*_2_	Proportion of diagnosed HIV-positive individuals with 200 < *l* < 350	0.2939	—	[10]
*ξ*_1_	Progression rate from *I*_1_ to *I*_2_	1/6	—	[10]
*ξ*_2_	Progression rate from *I*_2_ to *A*	1/3	—	[10]
*η*_1_	Progression rate from *T*_1_ to *T*_2_	1/12	—	[12]
*η*_2_	Progression rate from *T*_2_ to *T*_*A*_	1/6	—	[12]
*τ*_1_	Antiviral therapy coverage rate for HIV-positive individuals with *l* > 350	0	—	—
*τ*_2_	Antiviral therapy coverage rate for HIV-positive individuals with 200 < *l* < 350	0.2	—	[13, 14]
*α*_*I*_	Disease-related death rate for HIV infected individuals without receiving antiviral therapy	0.172	—	[15]
*α*_*T*_	Disease-related death rate for HIV infected individuals with antiviral therapy	0.06	—	[15, 16]
*α*_*A*_	Disease-related death for AIDS patients with antiviral therapy	0.136	—	[16, 17]
*S*(0)	Initial value of *S* (high-risk susceptible)	763240	87285	MCMC
*I*(0)	Initial value of *I* (individuals living with undiagnosed HIV)	5988	1274	MCMC
*I*_1_(0)	Initial value of *I*_1_ (HIV-positive individuals with *l* > 350)	99	—	Database
*I*_2_(0)	Initial value of *I*_2_ (HIV-positive individuals with 200 < *l* < 350)	49	—	Database
*A*(0)	Initial value of *A* (AIDS patients diagnosed at onset of AIDS symptoms)	53	—	Database
*T*_1_(0)	Initial value of *T*_1_ (HIV-positive individuals with *l* > 350 and receiving therapy)	0	—	Database
*I*_2_(0)	Initial value of *T*_2_ (HIV-positive individuals with 200 < *l* < 350 and receiving therapy)	0	—	Database
*T*_*A*_(0)	Initial value of *T*_*A*_ (AIDS patients diagnosed before onset of AIDS symptoms)	0	—	Database
*R*_0_	The basic reproduction number	3.8840	0.097	Calculated

## Appendix

### A. Parameter Estimation

Since the number of susceptible cases is large compared to the number of HIV/AIDS infected cases, *S*/*N* approximately equals 1. Thus we can get the following reduced model:

(A.1) $$\begin{array}{c} I^{\prime }=\tilde{\lambda }\left(t\right)-\left(d+\alpha_{I}+\delta \right)I,\\ I_{1}^{\prime }=\rho_{1}\delta I-\left(d+\alpha_{I}+\tau_{1}+\xi_{1}\right)I_{1},\\ I_{2}^{\prime }=\rho_{2}\delta I+\xi_{1}I_{1}-\left(d+\alpha_{I}+\tau_{2}+\xi_{2}\right)I_{2},\\ A^{\prime }=\left(1-\rho \right)\delta I+\xi_{2}I_{2}-\left(d+\alpha_{A}\right)A,\\ T_{1}^{\prime }=\tau_{1}I_{1}-\left(d+\alpha_{T}+\eta_{1}\right)T_{1},\\ T_{2}^{\prime }=\tau_{2}I_{2}+\eta_{1}T_{1}-\left(d+\alpha_{T}+\eta_{2}\right)T_{2},\\ T_{A}^{\prime }=\eta_{2}T_{2}-\left(d+\alpha_{A}\right)T_{A}, \end{array}$$

where $\tilde{\lambda }(t)=\beta c(1-\pi )(I+\varepsilon_{I_{1}}I_{1}+\varepsilon_{I_{2}}I_{2}+\varepsilon_{T_{1}}T_{1}+\varepsilon_{T_{2}}T_{2}+\varepsilon_{A}(A+T_{A}))$ and *ρ* = *ρ*_1_ + *ρ*_2_.

For ensuring good convergence of Markov Chain, some prior information is given. Xiao et al. [15] built a mathematical model which did not consider the different transmission routes and estimated several parameters involved in the model. They got the transmission coefficient is 0.386, the diagnose rate for HIV-positive individuals to be 0.304, and the proportion of HIV-positive individuals when diagnosed to be 0.864. Now we use them as the prior information for our estimation. That is to say, the initial values for *β*_0_, *δ*, and *ρ* are 0.386, 0.304, and 0.864, respectively. What is more, the ranges for each parameter and initial value are given: *β*_0_ ∈ (0,1), *δ* ∈ (0.01,0.5), *ρ* ∈ (0,1), *ε*_*T*_2__ ∈ (0.3,0.4177), *ε*_*A*_ ∈ (0,0.5), and *I*(0)∈(1000,8000). Here we assume *ε*_*T*_1__ = *ε*_*T*_2__ since there are no significant differences for the infectiousness of people in these two groups. Let $\tilde{I}(t)$ and $\tilde{A}(t)$ denote the real data, the number of annual reported HIV-positive individuals and AIDS patients from year 2005 to 2009, where *t* = 2005,2006,…, 2009. The proposal density is chosen to be multivariate normal distribution. Let *θ* = (*β*_0_, *δ*, *ρ*, *ε*_*A*_, *I*(0)), and *f*_1_(*θ*, *t*) denotes the numerical solution of the first equation of reduced model (A.1) at time *t*. Then the real value of $\tilde{I}(t)$ and $\tilde{A}(t)$ can be taken as random variables from Gaussian distributions with mean *ρδf*_1_(*θ*, *t*) and (1 − *ρ*)*δf*_1_(*θ*, *t*), respectively. During the MCMC simulation, we will minimize *ss* = ∑_*t*=2005_^*t*=2009^(*I*(*t*) − *ρδf*_1_(*θ*, *t*))^2^ + (*A*(*t*)−(1 − *ρ*)*δf*_1_(*θ*, *t*))^2^. We use a small MCMC package to achieve the estimation which provided in http://www.helsinki.fi/~mjlaine/mcmc/.

By fitting this reduced model to the annual reported HIV/AIDS cases from 2005 to 2009 we get the estimates of parameters *β*_0_, *ρ*, *δ*, *ε*_*T*_2__, *ε*_*T*_*A*__, and *I*(0) and their standard deviations. Based on these parameters, *U* and *S*(0) are estimated by fitting model (1) to the data.

### B. Basic Reproduction Number *R*_0_

The model has a disease-free equilibrium *E*_0_ = (*S*_0_, 0,0, 0,0, 0,0), where *S*_0_ = *U*/(*d* + *μ*), which can be obtained easily by setting the right side of model (1) to zero. Following the next-generation operator method of [28] we can get *R*_0_ of system (1). Let *β*_0_ = *βc*(1 − *π*), *ω*_1_ = *d* + *α*_*I*_ + *δ*, *ω*_2_ = *d* + *α*_*I*_ + *τ*_1_ + *ξ*_1_, *ω*_3_ = *d* + *α*_*I*_ + *τ*_2_ + *ξ*_2_, *ω*_4_ = *d* + *α*_*A*_, *ω*_5_ = *d* + *α*_*T*_ + *η*_1_, and *ω*_6_ = *d* + *α*_*T*_ + *η*_2_; then

(B.1) $$\begin{array}{c} F=\left(\begin{array}{ccccccc} \beta_{0} & \beta_{0}\varepsilon_{I_{1}} & \beta_{0}\varepsilon_{I_{2}} & \beta_{0}\varepsilon_{A} & \beta_{0}\varepsilon_{T_{1}} & \beta_{0}\varepsilon_{T_{2}} & \beta_{0}\varepsilon_{A}\\ 0 & 0 & 0 & 0 & 0 & 0 & 0\\ 0 & 0 & 0 & 0 & 0 & 0 & 0\\ 0 & 0 & 0 & 0 & 0 & 0 & 0\\ 0 & 0 & 0 & 0 & 0 & 0 & 0\\ 0 & 0 & 0 & 0 & 0 & 0 & 0\\ 0 & 0 & 0 & 0 & 0 & 0 & 0 \end{array}\right),\\ V=\left(\begin{array}{ccccccc} \omega_{1} & 0 & 0 & 0 & 0 & 0 & 0\\ -\rho_{1}\delta & \omega_{2} & 0 & 0 & 0 & 0 & 0\\ -\rho_{2}\delta & -\xi_{1} & \omega_{3} & 0 & 0 & 0 & 0\\ -\left(1-\rho \right)\delta & 0 & -\xi_{2} & \omega_{4} & 0 & 0 & 0\\ 0 & -\tau_{1} & 0 & 0 & \omega_{5} & 0 & 0\\ 0 & 0 & -\tau_{2} & 0 & -\eta_{1} & \omega_{6} & 0\\ 0 & 0 & 0 & 0 & 0 & -\eta_{2} & \omega_{4} \end{array}\right), \end{array}$$

such that

(B.2) R0=ρ(FV−1)=β0ω1[1+ρ1δεI1ω2+ρ1δξ1εI2ω2ω3+ρ2δεI2ω3      +ρ1δξ1ξ2εAω2ω3ω4+ρ2δξ2εAω3ω4+(1−ρ)δεAω4      +ρ1δτ1εT1ω2ω5+ρ1δτ1η1εT2ω2ω5ω6+ρ1δτ2ξ1εT2ω2ω3ω6+τ2ρ2δεT2ω3ω6      + ρ1δτ1η1η2εAω2ω5ω6ω4+ρ1δξ1τ2η2εAω2ω3ω6ω4+ρ2δτ2η2εAω3ω6ω4].

### C. Theoretical Analysis of System (1)

#### C.1. DFE Is Globally Attractive When *R*_0_ < 1

Proof Since *S* ≥ *N*, so *λ*(*t*) ≤ *βc*(1 − *π*)[*I* + *ε*_*I*_1__ *I*_1_ + *ε*_*I*_2__ *I*_2_ + *ε*_*T*_1__ *T*_1_ + *ε*_*T*_2__ *T*_2_ + *ε*_*A*_(*A* + *T*_*A*_)]; then, *I*′ ≤ *β*_0_[*I* + *ε*_*I*_1__ *I*_1_ + *ε*_*I*_2__ *I*_2_ + *ε*_*T*_1__ *T*_1_ + *ε*_*T*_2__ *T*_2_ + *ε*_*A*_(*A* + *T*_*A*_)] − *ω*_1_ *I*. Then we get the comparison system of system (1):

(C.1) x1′=β0[x1+εI2x2+εI2x3+εT1x5+ εT2x6+εA(x4+x7)]−ω1x1,x2′=ρ1δx1−ω2x2,x3′=ρ2δx1+ξ1x2−ω3x3,x4′=(1−ρ)δx1+ξ2x3−ω4x4,x5′=τ1x2−ω5x5,x6′=τ2x3+η1x5−ω6x6,x7′=η2x6−ω4x7.

Construct the Lyapunov function as

(C.2) V=1β0x1+(εI1ω2+εI2ξ1ω2ω3+εAξ1ξ2ω2ω3ω4  +εT2τ2ξ1ω2ω3ω6+εAη2τ2ξ1ω2ω3ω6ω4  + εT1τ1ω2ω5+εT2η1τ1ω2ω5ω6+εAη1η2τ1ω2ω5ω6ω4)x2+(εI2ω3+εAξ2ω3ω4+εT2τ2ω3ω6+εAη2τ2ω3ω6ω4)x3+εAω4x4+(εT1ω5+εT2η1ω5ω6+εAη1η2ω5ω6ω4)x5+(εT2ω6+εAη2ω6ω4)x6+εAω4x7.

What is more,

(C.3) $$\begin{array}{c} V^{\prime }=\left(\frac{R_{0}\omega_{1}}{\beta_{0}}-\frac{\omega_{1}}{\beta_{0}}\right)=\left(R_{0}-1\right)\frac{\omega_{1}}{\beta_{0}}x_{1}. \end{array}$$

It is easy to see that, when *R*_0_ < 1, we have *V*′ ≤ 0, if and only if *x*_1_ = 0, *V*′ = 0. That is to say system (C.1)'s largest invariant set is *F* = {*x*_1_ = 0}. According to Lasalle invariant set principle, when *t* → 0, solutions of system (C.1) satisfy *x*_1_ → 0. Then we get the terminal system of system (C.1):

(C.4) x2′=−ω2x2,x3′=ξ1x2−ω3x3,x4′=ξ2x3−ω4x4,x5′=τ1x2−ω5x5,x6′=τ2x3+η1x5−ω6x6,x7′=η2x6−ω4x7.

Obviously, all the eigenvalues of (C.4) are negative. Since system (C.4) is a linear differential system with constant coefficients, so the equilibrium (0,0, 0,0, 0,0, 0) is globally asymptotic stable. Then, the solutions of system (C.1) satisfy *x*_1_ → 0, *x*_2_ → 0, *x*_3_ → 0, *x*_4_ → 0, *x*_5_ → 0, *x*_6_ → 0, *x*_7_ → 0. From the comparison principle, we get that the disease-free equilibrium of system (1) is globally attractive.

#### C.2. The Uniform Persistence of the System

Proof of Theorem 2
Let *X* = *R*_+_^8^, *X*_0_ = *R*_+_ × Int(*R*_+_^7^), and ∂*X*_0_ : = *X*∖*X*_0_. Define a map *φ* : *R*_+_^8^ → *R*_+_^8^, which satisfies *φ*(*X* ^0^) = *u*(*ω*, *X* ^0^), where *u*(*t*, *X* ^0^) is the unique solution starting from the initial value *u*(0, *X* ^0^) = *X* ^0^. That is to say, *φ* is the solution semiflow. Firstly, we show that *φ* is uniformly persistent with respect to (*X*_0_, ∂*X*_0_). It is easy to see that *X* and *X*_0_ are positively invariant and the solutions of system (1) are uniformly and ultimately bounded. Thus the semi-flow is point dissipative on *R*_+_^8^, and *p* : *R*_+_^8^ → *R*_+_^8^ is compact. By [7], it follows that *φ* admits a global attractor. Define *M*_∂_ = {(*S*_0_, *I*_0_, *I*_1_0__, *I*_2_0__, *A*_0_, *T*_1_0__, *T*_2_0__, *T*_*A*_0__)∈∂*X*_0_ : *φ*(*S*_0_, *I*_0_, *I*_1_0__, *I*_2_0__, *A*_0_, *T*_1_0__, *T*_2_0__, *T*_*A*_0__)∈∂*X*_0_,  for  all*m* ≥ 0}. Now we will verify that

(C.5) $$\begin{array}{c} M_{\partial }=\left\{\left(S,0,0,0,0,0,0,0\right):S\geq 0\right\}. \end{array}$$

In fact, it is obvious that {(*S*, 0,0, 0,0, 0,0, 0) : *S* ≥ 0}⊆*M*_∂_, for any (*S*_0_, *I*_0_, *I*_1_0__, *I*_2_0__, *A*_0_, *T*_1_0__,  *T*_2_0__, *T*_*A*_0__)∈∂*X*_0_∖{(*S*, 0,0, 0,0, 0,0, 0) : *S* ≥ 0}.

1. If *I*_0_ > 0, then at least one of *I*_1_0__, *I*_2_0__, *A*_0_, *T*_1_0__, *T*_2_0__, and *T*_*A*_0__ is equal to zero. Let us assume that *I*_1_0__ = 0, then ${\dot{I}}_{1}(0)=\rho_{1}\delta I(0)>0$. Similarly, when anyone of *I*_2_0__, *A*_0_, *T*_1_0__, *T*_2_0__, and *T*_*A*_0__ is equal to 0, we can get ${\dot{I}}_{2}(0),\dot{A}(0),{\dot{T}}_{1}(0),{\dot{T}}_{2}(0),{\dot{T}}_{A}(0)>0$.
2. If *I*_0_ = 0, then at least one of *I*_1_0__, *I*_2_0__, *A*_0_, *T*_1_0__, *T*_2_0__, *T*_*A*_0__ is greater than 0. Let us assume that *I*_1_0__ > 0, then $\dot{I}(0)\geq \beta_{0}S(0)(\varepsilon_{I_{1}}I_{1}\left(0\right)/N\left(0\right))>0$, when any one of *I*_2_0__, *A*_0_, *T*_1_0__, *T*_2_0__, *T*_*A*_0__ is greater than 0, we can get the similar conclusion.

According to the above verification, we can get that for *t* > 0 sufficiently small, we have (*S*(*t*), *I*(*t*), *I*_1_(*t*), *I*_2_(*t*), *A*(*t*), *T*_1_(*t*), *T*_2_(*t*),  *T*_*A*_(*t*)) ∉ ∂*X*_0_; then (*S*(*t*), *I*(*t*), *I*_1_(*t*), *I*_2_(*t*), *A*(*t*), *T*_1_(*t*), *T*_2_(*t*),  *T*_*A*_(*t*)) ∉ *M*_∂_; that is to say, for any (*S*_0_, *I*_0_, *I*_1_0__, *I*_2_0__, *A*_0_, *T*_1_0__, *T*_2_0__, *T*_*A*_0__) ∉{(*S*, 0,0, 0,0, 0,0, 0) : *S* ≥ 0} there must be (*S*_0_, *I*_0_, *I*_1_0__, *I*_2_0__, *A*_0_, *T*_1_0__, *T*_2_0__, *T*_*A*_0__) ∉ *M*_∂_; then *M*_∂_⊆{(*S*, 0,0, 0,0, 0,0, 0) : *S* ≥ 0}. So, *M*_∂_ = {(*S*, 0,0, 0,0, 0,0, 0) : *S* ≥ 0}. Obviously, *E*_0_(*S*_0_, 0,0, 0,0, 0,0, 0) is a fixed point of *φ* in *M*_∂_. If (*S*(*t*), *I*(*t*), *I*_1_(*t*), *I*_2_(*t*), *A*(*t*),  *T*_1_(*t*), *T*_2_(*t*), *T*_*A*_(*t*)) is a solution initiating from *M*_∂_, then when *t* → *∞*, we have *S*(*t*) → *S*_0_, *I*(*t*) → 0, *I*_1_(*t*) → 0, *I*_2_(*t*) → 0, and *A*(*t*) → 0, *T*_1_(*t*) → 0, *T*_2_(*t*) → 0, *T*_*A*_(*t*) → 0. Since every solution initiating from *M*_∂_ will stay in *M*_∂_ forever, so *E*_0_ is an isolated invariance. Since *R*_0_ > 1, there exists a *ε* small enough such that

(C.6) $$\begin{array}{c} 1-\frac{S^{*}-\varepsilon }{S^{*}+8\varepsilon }R_{0}<0. \end{array}$$

The following proof is that *W* ^*s*^(*E*_0_)∩*X*_0_ = *ϕ*. That is to say, there exists a positive constant *δ*_0_, such that for any solution *u*(*t*, *X* ^0^) initiating from *X*_0_, lim⁡_*t*→*∞*_sup⁡||*u*(*t*, *X* ^0^) − *E*_0_|| ≥ *δ*_0_. Suppose that for any positive *δ*_0_ (let *δ*_0_ = *ε*),

(C.7) $$\begin{array}{c} \underset{t\to \infty }{\lim}\sup||u\left(t,X^{0}\right)-E_{0}||<\varepsilon . \end{array}$$

Then, there exists *t*_0_ > 0 large enough such that when *t* > *t*_0_, *S*_0_ − *ε* < *S*(*t*) < *S*_0_ + *ε*, *I*(*t*) < *ε*, *I*_1_(*t*) < *ε*, *I*_2_(*t*) < *ε*, *A*(*t*) < *ε*, *T*_1_(*t*) < *ε*, *T*_2_(*t*) < *ε*, *T*_*A*_(*t*) < *ε*. It follows that *S*_0_ − *ε* < *N*(*t*) < *S*_0_ + 8*ε*. What is more, we have

(C.8) I′≥β0·(S0−ε)×I+εI1I1+εI2I2+εAA+εT1T1+εT2T2+εATAS0+8ε−ω1I.

Now we get the comparison system:

(C.9) x1′=β0·(S0−ε)×x1+εI1x2+εI2x3+εAx4+εT1x5+εT2x6+εAx4S0+8ε−ω1x1,x2′=ρ1δx1−ω2x2,x3′=ρ2δx1+ξ1x2−ω3x3,x4′=(1−ρ)δx1+ξ2x3−ω4x4,x5′=τ1x2−ω5x5,x6′=τ2x3+η1x5−ω6x6,x7′=η2x6−ω4x7.

The characteristic equation of (C.9) is

(C.10) Q=λ7+a1λ6+a2λ5+a3λ4+a4λ3+a5λ2+a6λ1+a7,

where *a*_7_ = *ω*_1_ *ω*_2_ *ω*_3_ *ω*_4_ *ω*_5_ *ω*_6_ *ω*_4_(1 − *R*_0_((*S*_0_ − *ε*)/(*S*_0_ + 8*ε*))) < 0. Since *λ* ^1^ *λ* ^2^ *λ* ^3^ *λ* ^4^ *λ* ^5^ *λ* ^6^ *λ* ^7^ = −*a*_7_ > 0, so it has at least one of positive real eigenvalues. Let us assume that *λ** > 0, (*x*_1_, *x*_2_, *x*_3_, *x*_4_, *x*_5_, *x*_6_, *x*_7_) is the eigenvector. Suppose *x*_1_ > 0; then *x*_2_ = *ρ*_1_ *δx*_1_/(*λ** + *ω*_2_) > 0, *x*_3_ = (*ρ*_2_ *δx*_1_ + *ξ*_1_ *x*_2_)/(*λ** + *ω*_3_) > 0, *x*_4_ = ((1 − *ρ*)*δx*_1_ + *ξ*_2_ *x*_3_)/(*λ** + *ω*_4_) > 0, *x*_5_ = *τ*_1_ *x*_2_/(*λ** + *ω*_5_) > 0, *x*_6_ = (*τ*_2_ *x*_3_ + *η*_1_ *x*_5_)/(*λ** + *ω*_6_) > 0, and *x*_7_ = *η*_2_ *x*_6_/(*λ** + *ω*_7_) > 0. That is to say there exists a positive real eigenvalue which has a positive eigenvector. This implies that the solution tends to infinity as *t* → *∞*, which contradicts (C.7). This completes the proof.

### D. The Proof for *B* > 0

Proof Suppose *ρ*_1_ = *kρ*_2_, *ξ*_2_ = *kξ*_1_, where *k* > 1. Since *ξ*_1_ < *ξ*_2_, so *ω*_2_ < *ω*_3_. Meanwhile, *η*_1_ < *ξ*_1_ < *ξ*_2_ and *α*_*T*_ < *α*_*I*_, so *ω*_5_ < *ω*_3_. Consequently, 1/*ω*_2_ > 1/*ω*_3_ and 1/*ω*_5_ > 1/*ω*_3_. Then we have

(D.1) Bρ1δ=η1(d+αI+ξ1)ω22ω5ω6+d+αI+ξ2kω32ω6 +ξ1ω2ω3ω6−ξ1τ(ω2+ω3)ω22ω32ω6>η1(d+αI+ξ1)ω22ω3ω6+d+αI+ξ2kω32ω6+ξ1ω2ω3ω6 −ξ1τω2ω32ω6−ξ1τω22ω3ω6>η1(d+αI+ξ1)ω22ω3ω6+d+αI+ξ2kω32ω6−ξ1τω2ω32ω6=1ω3ω6[η1(d+αI+ξ1)ω22+d+αI+ξ2kω3−ξ1τω2ω3].

We only need to verify that (*η*_1_(*d* + *α*_*I*_ + *ξ*_1_)/*ω*_2_^2^)+((*d* + *α*_*I*_ + *ξ*_2_)/*kω*_3_)−(*ξ*_1_ *τ*/*ω*_2_ *ω*_3_) > 0. Reducing it to a common denominator, we get the numerator which is denoted by $\bar{\theta }$. We just verify that $\bar{\theta }>0$:

(D.2) θ¯=kη1(d+αI+ξ1)(d+αI+ξ2τ) +(d+αI+ξ1+τ)2(d+αI+ξ2)−kξ1τ(d+αI+ξ1τ)=kη1(d+αI+ξ1)(d+αI+ξ2+τ) +(d+αI+ξ1+τ)2(d+αI+ξ2)−kξ1τ(d+αI+ξ1τ)=kη1(d+αI)2+kη1(ξ1+ξ2+τ)(d+αI) +kη1ξ1(ξ2+τ)+(d+αI+ξ1+τ)2(d+αI) +(d+αI+ξ1+τ)2ξ2 −kξ1τ(d+αI)−kξ12τ−kξ1τ2=kη1(d+αI)(d+αI+ξ1+ξ2+τ) +(d+αI+ξ1+τ)2(d+αI)+kη1ξ1(ξ2+τ) +(d+αI)2ξ2+2ξ2(ξ1+τ)(d+αI) +ξ2(ξ1+τ)2−kξ1τ(d+αI)−kξ12τ−kξ1τ2=kη1(d+αI)(d+αI+ξ1+ξ2+τ) +(d+αI+ξ1+τ)2(d+αI)+ξ2(d+αI)2 +(2kξ12+2kξ1τ−kξ1τ)(d+αI) +kξ13+2kξ12τ+kξ1τ2+k2ξ12η1 +kξ1η1τ−kξ12τ−kξ1τ2=kη1(d+αI)(d+αI+ξ1+ξ2+τ) +(d+αI+ξ1+τ)2(d+αI)+ξ2(d+αI)2 +(2kξ12+2kξ1τ−kξ1τ)(d+αI) +kξ13+kξ12τ+k2ξ12η1+kξ1η1τ>0.

This completes the proof.
